# Rapamycin-Loaded, Capryol^TM^ 90 and Oleic Acid Mediated Nanoemulsions: Formulation Development, Characterization and Toxicity Assessment

**Published:** 2018

**Authors:** Hamideh Sobhani, Parastoo Tarighi, Seyed Nasser Ostad, Alireza Shafaati, Nastaran Nafissi-Varcheh, Reza Aboofazeli

**Affiliations:** a *Department of Pharmaceutics, School of Pharmacy, Shahid Beheshti University of Medical Sciences, Tehran, Iran. *; b *Department of Medical Biotechnology, Faculty of Allied Medicine, Iran University of Medical Sciences, Tehran, Iran. *; c *Department of Pharmacology and Toxicology, Faculty of Pharmacy, Tehran University of Medical Sciences, Tehran, Iran.*; d *Department of Pharmaceutical Chemistry, School of Pharmacy, Shahid Beheshti University of Medical Sciences, Tehran, Iran. *; e *Department of Pharmaceutical Biotechnology, School of Pharmacy, Shahid Beheshti University of Medical Sciences, Tehran, Iran.*; f *Department of Pharmaceutics, School of Pharmacy and Protein Technology Research Center, Shahid Beheshti University of Medical Sciences, Tehran, Iran.*

**Keywords:** Rapamycin, Nanoemulsion, Phase diagram, MTT, TEER

## Abstract

This study was planned to explore the capability of nanoemulsions (NEs) consisting of Capryol^TM ^90 and oleic acid for the delivery of rapamycin (RAP). Permeability and cytotoxicity of RAP-loaded NEs were also inspected. Pseudo-ternary phase diagrams were created with oleic acid and Capryol^TM ^90 (as oil phase) and four surfactants and co-surfactants at various weight ratios (R_sm_). Selected NEs from O/W region on the phase diagrams with the drug concentration of 1 mg/mL, were prepared via the spontaneous emulsification technique, characterized for particle size and subjected to stability tests at various temperatures over 9-12 months. Cumulative drug release was determined for a period of 48 h using a dialysis sac. The assay of RAP was determined using HPLC technique. Cytotoxicity of NEs was evaluated by MTT assay on breast cancer cell line, namely SKBR-3. The permeability of RAP-loaded NEs across Caco-2 monolayers was assessed by measurement of TEER (transepithelial electrical resistance) value. The intracellular uptake of coumarin 6-loaded NEs by SKBR-3 cells was also investigated using florescence microscopy. NEs containing oleic acid/Tween 20/propylene glycol, Capryol^TM^ 90/Tween 20/*iso*-propanol, and Capryol^TM^ 90/Cremophor^®^ RH40/Transcutol^® ^P showed more cytotoxicity and permeability compared with the RAP methanolic solution. The minimum toxic concentration of RAP in NE formulations was found to be 7.5 µg/mL. The highest intracellular uptake was observed for the NE composed of Capryol^TM^ 90/Tween 20/*iso*-propanol which was in consistent with the results obtained from cytotoxicity and permeability tests. The overall results implicated that this novel carrier was effective for enhancing RAP permeation in Caco-2 cell membrane along with enhancement of cytotoxicity.

## Introduction

Rapamycin (RAP), also identified as sirolimus, is an antibiotic from macrolide family with both immunosuppressant and anti-tumor properties. It is extracted from *Streptomyces hygroscopicus*. It works by inhibition of the mammalian target of rapamycin (mTOR), as a member of protein kinase family. mTOR could control several cellular processes, comprising of cell growth, cell proliferation, as well as transcription and synthesis of protein. Previous investigations have pointed to mTOR as an ideal target for anti-cancer agents. RAP inhibits tumor growth by preventing tumor cell apoptosis, and angiogenesis. It suppresses immune actions by inhibition of T and B cell proliferation with the same mechanisms (1-4).

Despite the promising pharmacological activities, at the same time, RAP possesses some improper characteristics. RAP is an intensely hydrophobic drug and practically insoluble in water (2.6 µg/mL). Its sensitivity to gastric acid and high hepatic first-pass metabolism results in limited intestinal absorption and low oral bioavailability, respectively. On the other hand, this lipophilic drug is the substrate of CYP450 3A enzymes and P-glycoprotein (P-gp) efflux pump, present at cell membrane. These characteristics have been considered as significant factors that contribute to low oral bioavailability (5-13). In addition, RAP shows limited distribution to the site of action as a consequence of intensely partition into the erythrocytes (14). 

Systemic administration of RAP could also produce a sum of adverse effects which could affect its therapeutic effectiveness. Extensive distribution in many organs, a very long half-life and a strong immunosuppressive effect following the systemic administration limit the long-term clinical application of RAP (15). RAP poor solubility in some common parenteral solvents (*e.g.*, ethanol, propylene glycol, propylene glycol) and the lack of any functional group with the ability to ionize in the pH range of 1-10, have consequently made the preparation of injectable formulations difficult (11, 16 and 17).

There are various methods available to formulate poorly water soluble drugs. Many investigations have been conducted to develop new delivery systems for RAP, mainly in an attempt to increase the solubility and dissolution properties, to improve bioavailability and loading of drug, to increase the drug protection and stability, and to provide drug targeting and extended release pattern. Some examples are the use of poly(lactic-co-glycolic)acid (PLGA) microparticles (18, 19), poly(l-lactide-co-caprolactone-co-glycolide) (PLCG) nanofibers (20), chitosan/polylactic acid nanoparticles (21), liposomes (16), amphiphilic co-polymer micelles of poly(ethylene glycol)-b-poly(epsilon-caprolactone) (PEG-PCL) (17) and the application of solid dispersion and complexation techniques with different hydrophilic excipients (22). 

Nanoemulsion (NE) is a kind of lipid-based formulation that has been the focus of much research due to its potential advantages from the pharmaceutical viewpoint. This self-emulsifying system is considered as a desirable carrier for improving the bioavailability of drugs with low water-solubility. Generally accepted, NEs are dispersions of oil and water with transparent or translucent appearance, stabilized by an interfacial layer composed of combined surfactant and co-surfactant molecules, with droplet size in the range of 50-200 nm. It has been reported in the literature that drug delivery by NEs is advantageous for some reasons; a) they have ultralow interfacial tensions and possess a relatively high kinetic stability, with no apparent droplet flocculation and coalescence (23, 24) (therefore, they are sometimes described as “Approaching Thermodynamic Stability”) (25); b) the large interfacial areas associated with the presence of nano-droplets would affect the drug transport property (26); c) high solubilization capacity compared to simple micellar solutions; d) their long term physical stability offers advantages over unstable dispersions; e) they are easily prepared; f) they improve the solubility and the mucosal permeability of lipophilic compounds (27, 28); and g) they protect the sensitive drugs from hydrolysis and enzymatic degradations in physiologic environments (29-32). 

The main aim of this study was to attain an effective delivery system for RAP. It was hypothesized that by loading RAP in NEs, its bioavailability and anti-proliferative effect would increase. In the previous study, we developed and assessed the toxicity of triacetin-mediated NEs (33). The present investigation was planned to formulate and characterize Capryol^TM^ 90 and oleic acid-based NEs. Capryol^TM^ 90 and oleic acids were chosen as the oil phase for the drug solubilization. Finally, NEs permeability across Caco-2 cell monolayer and their cytotoxicity on SKBR-3 cell line were also evaluated. 

## Experimental

*Materials*

Tween 80, Tween 20, triacetin, *iso*-propanol, polyethylene glycol 400 (PEG 400), propylene glycol (PG) and methanol were purchased from Merck Chemical Co. (Germany). Labrasol (caprylocaproyl macrogol-8-glycerides) and Transcutol^® ^P (diethylene glycol monoethyl ether) were gifted by Gattefosse Co. (France). Cremophor RH 40 was provided as a gift from Osvah Pharmaceutical Co. (Tehran, Iran). Penicillin-streptomycin (Pen strep) 100X and RPMI 1640 medium were provided from Biosera (England). Dulbecco’s Modified Eagle’s Medium (DMEM-high glucose), fetal bovine serum (FBS), trypsin-ethylenediamine tetraacetic acid (EDTA), 4-(2-hydroxyethyl)-1-piperazineethanesulfonic acid (HEPES) and Hank’s Balanced Salt Solution (HBSS) were prepared from PAA Laboratories GmbH (Austria). Trypan blue (0.4% w/v in PBS) and 3-(4,5-dimethylthiazol-2-yl)-2,5-diphenyl tetrazolium bromide (MTT) were provided from Sigma (USA) and DMEM F12 was obtained from Atocel (Austria). Purified water was collected from a Millipore Milli-Q plus Water Purification System (Millipore, France). Rapamycin (RAP; sirolimus, batch number SIRI0012) and Rapamune were purchased from Euticals Spa Co. (Italy), and Pfizer (Netherlands), respectively.

*Construction of pseudoternary phase diagrams*

Mixtures of surfactants (Tween 20, Tween 80, Labrasol^®^ and Cremophor^®^ RH 40) and co-surfactants (Transcutol P, *iso*-propanol, PEG and PG) at different surfactant/co-surfactant weight ratios (R_sm_; namely, 1:2, 1:1, 2:1) were prepared. Appropriate amounts of oleic acid or Capryol^TM^ 90 were mixed with the appropriate amounts of the surfactant mixture phase, in order to prepare oil-surfactant mixtures with three weight ratios (1:9 to 9:1). The samples were mixed in capped vials at room temperature till a transparent solution was prepared. For construction of phase diagrams, the samples were titrated with distilled water and agitated for a necessarily long time to attain the equilibrium. Each titration was checked both visually and through cross-polaroids for determining the clarity and birefringent liquid crystalline phase, respectively. Triangle phase diagrams were plotted with the three apices demonstrating a fixed R_sm_, oil and water contents. All mixtures, produced optically transparent or translucent, non-birefringent, isotropic solutions at low oil and low water domains were termed oil in water (o/w) and water in oil (w/o) NEs, respectively.

*High performance liquid chromatography*

The samples were assayed by a previously described HPLC method (34). HPLC analysis was implemented on Knauer HPLC system (Germany), comprising of a UV detector (Smartline 2500), pump (Smartline 1000), and software (Chromgate V3.1.7). Separation was done by using a reversed phase C8 column (MZ, 15 × 4.6 mm, 5 μm, MZ Analysentechnik GmbH, Germany). A suitable guard column was also used. A combination of methanol : water (80:20 v/v) was used as mobile phase that freshly prepared and degassed every day. Column temperature was fixed at 57 °C (Knauer, Germany). The injection of samples was performed on a Reodyne injector equipped with a 20 μL loop. UV detector was set at 277 nm. The method was confirmed for RAP assay with respect to specificity, linearity, precision (intra/inter-day RSD), and accuracy.

*Determination of rapamycin solubility in oleic acid and Capryol* ^TM^ *90*

The solubility of RAP in oleic acid and Capryol^TM^ 90 was assessed by addition of excess quantity of the drug in 1 mL of the each oil. The mixture was agitated at room temperature for 72 h to be equilibrated. The excess of the drug was removed and the sample was centrifuged for 15 min at 14000 rpm. The supernatant was filtered through 0.2 µm membrane filter. The solubility of RAP was then estimated by the above-mentioned HPLC method.

*Preparation of nanoemulsions containing rapamycin*

The type of components and their required concentrations which lead to the production of NEs were determined by construction of pseudo-ternary phase diagrams. Following the determination of o/w NE regions on the phase diagrams, those systems that indicated a relatively extended o/w NE domain and that allowed to choose an oil content for complete solubilization of the drug, were selected. Following the preparation of the selected blank NEs and measurement of their particle size, those systems with the particle size of less than 100 nm and polydispersity index (PDI) of less than 0.5 which remained transparent/translucent after 72 h storage at room temperature, were chosen for drug loading.

RAP-NEs were prepared using spontaneous emulsification technique. One mg of RAP was added to 1 mL of mixture containing oil/surfactant/co-surfactant with constant stirring, using a mechanical stirrer, until a transparent solution was obtained. Finally, the required amount of water was added and the mixture was agitated gently until a transparent/translucent NE was achieved.

*Measurement of particle size and zeta potential*

The size of particles, PDI, and zeta potential of all NEs containing RAP were measured, using Malvern Zetasizer (Nano-ZS, Malvern Instruments, Worcestershire, UK), with Nano-ZS software for data analysis. All analyses were implemented three 3 times.

*Transmission electron microscopy (TEM)*

To observe the two-dimensional, relative size morphology of RAP-loaded NE particles, a drop of the sample was directly deposited on the holey film grid and after drying, the images were taken with magnification of 11000, using a transmission electron microscope (Philips CM30, Netherlands).

*In-vitro drug release*

The profile of RAP release from the developed NEs was assessed by the dialysis bag method. Dialysis bags (MWCO 12 KD, Iran) were placed in distilled water for 24 h and kept refrigerated until usage. One mL of the drug-loaded NE (containing 1 mg RAP) was packed in a dialysis bag, which was then sealed with clips at both ends and placed in release medium (100 mL water having 0.05% w/v Tween 80). The assembly was stirred with speed of 100 rpm at 37 ± 1 °C and the aliquots of 2 mL were taken from the dissolution media at predetermined certain times (1, 2, 4, 8, 12, 24, 36 and 48 h). The same volume of fresh media was added to ensure the sink condition. The samples were evaluated for drug content using an HPLC method as described above.

*Stability tests*

Developed RAP-loaded NEs were subjected to stability test. The samples were stored at 4, 25, and 40 °C and also, their stability was checked over a period of 9-12 months. Phase separation, particle size, PDI, and drug content of NEs were then assessed.

*Cell culture*

Live SKBR-3 cells (National Cell Bank of Iran Code: 207) were purchased from Pasteur Institute of Iran (passage number: 8). The cells were cultured in 25 cm^2 ^plastic flasks (Nunc, Denmark) in an enriched media containing 84% v/v Dulbecco′s Modified Eagle’s Medium (DMEM high glucose), 15% v/v FBS and 1% v/v penicillin-streptomycin-100x (pen-strep) and incubated in a controlled moistened incubator having 5% CO_2_ and 95% air at 37 °C. The culture medium of flask was altered every two days. To passage the cells after reaching to 70-80% cell consistency, the cells were trypsinized with trypsin-EDTA (1x) and resuspended in the culture medium and kept at 37 °C. The cells with passage number up to 20 were used for cytotoxicity study.

For permeability study, Caco-2 cell line (passage number 40-50) was used. The cells were incubated at 37 °C in 75 cm^2^ plastic flasks in a controlled moistened atmosphere of 5% CO_2_ and 95% air, using 15 mL of a medium containing 50% v/v RPMI 1640, 34% v/v DMEM F12,15% v/v FBS and 1% v/v penicillin-streptomycin (100 IU/mL). Culture medium was replaced every two days. At final stage, the cells were trypsinized with trypsin-EDTA (1x), passaged and maintained for 7 days to reach the 80-90% confluency. The cells with a passage number up to 20 were used for cytotoxicity studies.

*Cytotoxicity assay*

Cytotoxicity of drug-free and drug-loaded NEs was evaluated, using MTT to assess the viability of cells (35). The cells at the density of 1 × 10^4^ viable SKBR-3 cells per well were cultured in 96-well microplate and incubated for 48 h to allow the cell attachment. The cells were incubated with blank NEs, RAP-loaded NEs, and RAP-methanolic solution with the final drug concentrations of 7.5, 10, and 15 nM, respectively, in an atmosphere of 5% CO_2_ at 37 °C for 48 h. For each concentration, three wells were used per plate. Two wells were allocated for negative (treated with medium only) and positive (treated with DMSO) controls. After washing with PBS, the cells remained in contact with 20 µL of MTT solution (5 mg/mL) at 37 °C. Following 4 h incubation, 100 µL of DMSO was then added to each well, while stirring vigorously, in order to dissolve formazon crystals. The optical absorbance of each well was measured using enzyme-linked immunosorbent assay reader (Anthous 2020; Anthos Labtec Instruments, Salzburg, Austria) at the wavelength of 550 nm. The cell viability was finally determined as a percentage of the negative control.

*Measurement of Transepithelial Electrical Resistance (TEER)*

Semipermeable polycarbonate filter inserts (Nunc 12-well transwell plates, pore size 0.4 µm, surface area 13 cm^2^) were used to seed Caco-2 cells. The filters were precoated with 100 µL of diluted rat tail collagen (type Ι) solution (in 0.1 N acetic acid with the ratio of 1:9) in an attempt to enhance the cell adhesion. Coated filter inserts were incubated overnight to become dried. The cells with density of 4 × 10^5^ cells/cm^2^ were then seeded on polycarbonate filters (36, 37). Transepithelial electrical resistance (TEER) of Caco-2 cell monolayer was measured for 21 days after seeding the cells on the filter to estimate the monolayer integrity. EVOM 2 voltohmmeter with chopstick electrodes (World Precision Instruments, Sarasota, USA) was employed for TEER measurement. Culture medium at both apical (500 µL) and basolateral sides (1500 µL) was changed every other day. Mean TEER values across the Caco-2 cell monolayers were measured with the formula below: 

TEER = (R_monolayer _- R_blank_) × A (Ω.cm^2^)

where R_monolayer_ indicates the resistance of Caco-2 monolayer, A is the available surface area of the filter inserts and *R*_blank _shows the resistance of inserts without Caco-2 monolayer. TEER values between 200-400 Ω.cm^2 ^show a good integrity of the cell monolayer. TEER evaluation were also performed during the permeation study at the specific times (0, 1, 2, 3, 4, 8 and 24 h) after the permeation tests in order to assess the effects of NEs on opening the tight junction barriers. 

*Transport study*

To conduct the transport tests, the cells were washed twice with PBS and incubated with transition buffer comprising HPSS (pH 7.4) supplemented with 25 mM HEPES for 30 min. After removing the transition buffer, RAP-loaded NEs and RAP methanolic solution were placed in the apical side of cell monolayer at the concentration of 1 mg/mL. Transition buffer (1.5 mL) was placed in the basolateral side of the monolayer. All transport analysis was performed at 37 °C from apical (A) to basolateral (B) direction of cell monolayer. The samples were taken at predetermined time intervals (0, 1, 2, 3 and 4 h) from the basolateral side and analyzed with HPLC method described above. The apparent permeability coefficient (P_app_) was finally calculated according to the formula below: 

P_app _= Q/(A × t × C_0_)

where Q is the cumulative amount of the drug in the receptor compartment, A represents the surface area of filter, t is the time of sampling and C_0_ indicates the primary drug concentration in the apical compartment.

*Cellular uptake of nanoemulsions*

In a 12-well plate, SKBR-3 cells were cultured with density of 40000 cells per well and incubated in the presence of NEs loaded with coumarin 6 and also methanolic solution of coumarin 6 (as the control) at a concentration of 0.5 µg/mL. After 6 h, the cells were washed with PBS and the cellular uptake of coumarin 6-loaded NEs was then evaluated using fluorescence microscopy. The intensity of fluorescent color in each obtained image was finally determined.

*Statistical analysis*

Data are reported as mean ± SD. Statistical study of differences between the samples was carried out using one-way ANOVA and an appropriate post-test, if necessary. The level of probability, 0.05, was considered as the level of significance.

## Results and Discussion

*Phase behavior studies*

**Figure 1:**
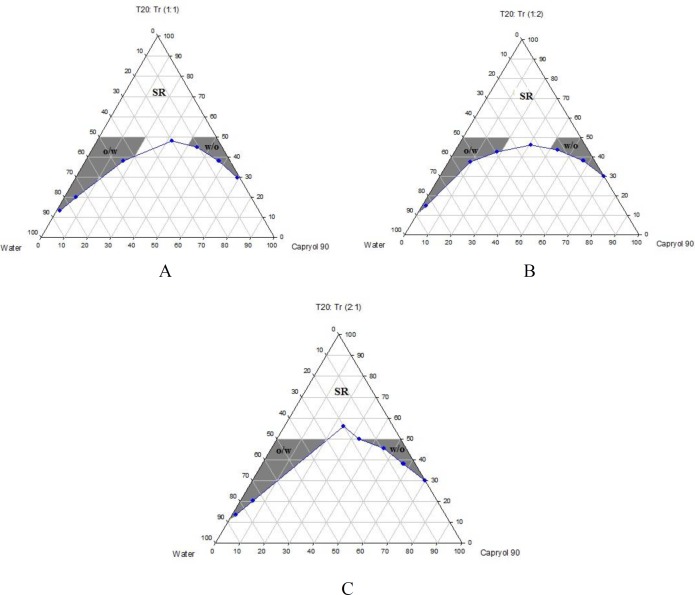
Phase diagrams of quaternary systems containing Capryol^TM^ 90/Tween 20: Transcutol  P/water at A) R_sm_ of 1:1; B) R_sm_ of 1:2; and C) R_sm_ of 2:1 (O/w, w/o and SR represent oil-in-water, water-in-oil and surfactant-rich areas, respectively).

**Figure 2:**
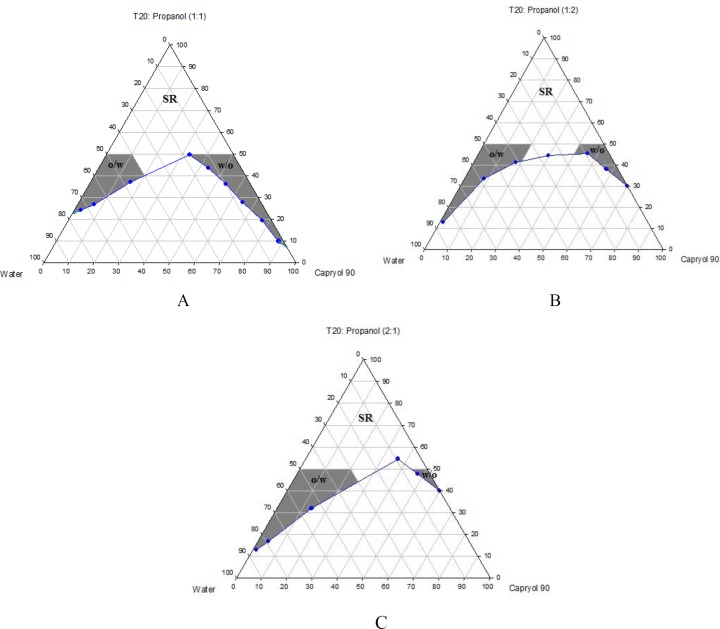
Phase diagrams of quaternary systems containing Capryol^TM^ 90/Tween 20: *iso*-propanol/water at A) R_sm_ of 1:1; B) R_sm_ of 1:2; and C) R_sm_ of 2:1 (O/w, w/o and SR represent oil-in-water, water-in-oil and surfactant-rich areas, respectively).

**Figure 3:**
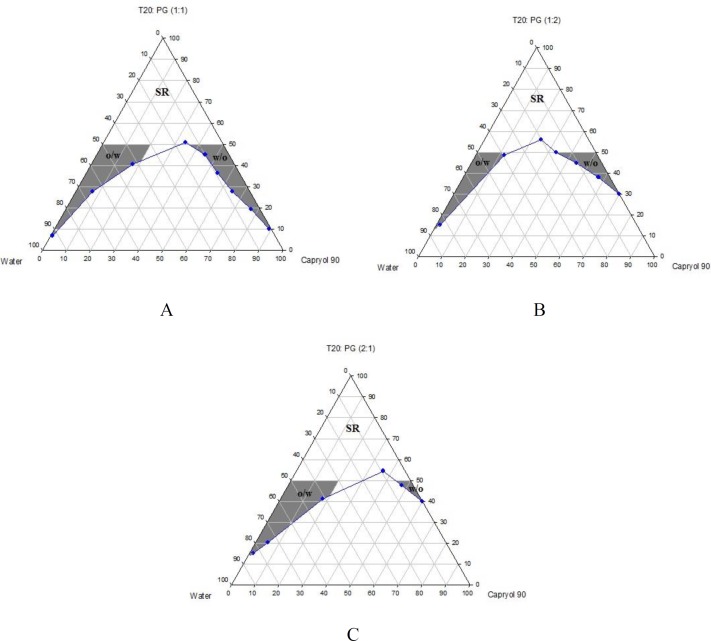
Phase diagrams of quaternary systems containing Capryol^TM^ 90/Tween 20/ PG/ water at A) R_sm_ of 1:1; B) R_sm_ of 1:2; and C) R_sm_ of 2:1 (O/w, w/o and SR represent oil-in-water, water-in-oil and surfactant-rich areas, respectively).

**Figure 4:**
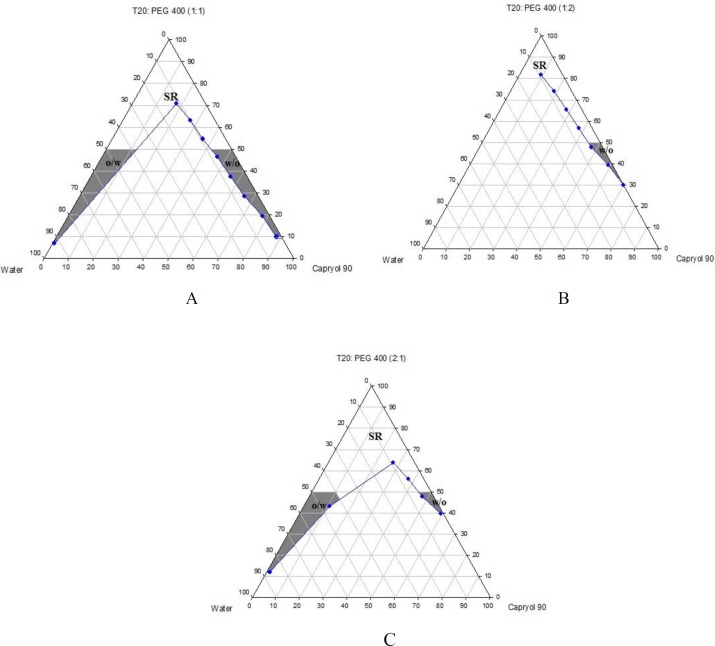
Phase diagrams of quaternary systems containing Capryol^TM^ 90/Tween 20/PEG 400/water at A) R_sm_ of 1:1; B) R_sm_ of 1:2; and C) R_sm_ of 2:1 (O/w, w/o and SR represent oil-in-water, water-in-oil and surfactant-rich areas, respectively).

Phase diagrams of systems comprising of two different oils (oleic acid and Capryol^TM^ 90), four different surfactants and co-surfactants at three R_sm _were constructed. Due to the small o/w NE area in the presence of oleic acid and because of the similarities between the phase diagrams constructed with Capryol^TM ^90, in order to avoid overcrowding of the report, only the phase diagrams of systems composed of Capryol^TM^ 90/Tween 20 are presented diagramatically. Wherever appropriate, any differences between phase behaviors observed with other surfactants are mentioned in the text. Figures 1-4 illustrate the diagrams of Capryol^TM^ 90/Tween 20 mixtures in the presence of all co-surfactants.

As can be seen, a mixture of Capryol^TM^ 90/Tween 20 can produce three different transparent areas, including a transparent/translucent domain in the water-rich region, a transparent domain in the oil-rich part and a surfactant–rich (SR) area, designated as o/w, w/o, and SRA, respectively (except for Tween 20/PEG 400/water at R_sm_ of 1:2), the extent of which depended upon the nature of co-surfactant and R_sm_. It should be noted that since accurate determination of the boundaries between the NE domains and SR region was difficult, the area with the extension up to 50% w/w surfactant mixture was considered as NE, above which the area was labeled as SR domain. Despite some differences between the phase diagrams, it is advantageous to mention the similarities. Generally, the following generalizations can be made about the systems studied:

*Capryol* ^TM^ *90 systems*

In most systems, regardless of the type of surfactant and co-surfactant, the change of R_sm_ did not affect the extent of NE area significantly.

Except for Labrasol^®^/*iso*-propanol and Tween 80/*iso*-propanol systems, all three o/w, w/o and SR domains were observed,

The extent of NE domain in the presence of co-surfactants followed the order of *iso*-propanol > Transcutol^® ^ P > PG > PEG 400.

Transparent o/w NE formulations were generally prepared with low viscosity. However, few systems were translucent especially at high water content. 

The extent of NE increased as the amount of surfactant mixture increased.

The largest o/w NE area was seen in the presence of Cremophor^®^ RH40 and Transcutol^® ^P, whereas the smallest domain was observed in the presence of Labrasol^®^ and PG.

No NE area was detected in formulations composed of Labrasol^®^, PEG 400 and Capryol^TM^ 90.

No liquid crystal phase was observed in all formulations.

None of the systems with less than 10 wt% total surfactant concentrations could solubilize water. 

*Oleic acid systems*

In most formulations, depending upon the type of the co-surfactant and R_sm_, w/o NE region with various extents was observed.

No o/w NE can be produced in the presence of PEG 400 as the co-surfactant.

Water and oil solubilizing capacities increased with increasing surfactant/co-surfactant content, irrespective of the R_sm_. 

By using PG as the co-surfactant, a very narrow o/w NE region was observed in a few systems, such as Labrasol^®^/PG (R_sm_ of 2:1) and Tween 20/PG (R_sm_ of 1:2).

Regardless of type of surfactant, *iso*-propanol seemed to be the most appropriate co-surfactant for producing o/w NEs.

In most formulations, a change of R_sm_ could affect the phase behavior.

No liquid crystal phase was observed in all formulations.

O/w NE formulations generally appeared to be transparent with low viscosity. However, systems composed of Tween 20, Tween 80, Labrasol and Cremophor^®^ RH 40 in the presence of *iso*-propanol at R_sm_ of 1:1 and 1:2 were translucent at high water content.

None of systems with less than 10 wt% total surfactant concentrations could solubilize water**.**

*Selection of o/w nanoemulsions from phase diagrams*

Based on the extent of o/w region on the constructed phase diagrams, o/w NEs were selected for drug incorporation, considering the minimum possible surfactant/co-surfactant concentration, allowing the access to the enough oil content for complete solubilization of RAP and achievement of stable RAP-loaded NEs without any drug precipitation. Among 48 systems containing oleic acid and 48 systems composed of Capryol^TM^ 90, only 10 and 13 formulations showed the desired features, respectively, and therefore they were selected for characterization studies concerning the particle size and clarity. Finally, those systems with particle size of less than 100 nm, PDI of less than 0.5 which were clear after 72 h were selected for drug loading (1 mg RAP/mL of NE) and further investigations. Table 1 presents the components of all blank NEs chosen from the phase diagrams and indicates those systems that were used for drug incorporation, at a fixed total surfactant concentration of 40% w/w.

Pharmaceutical acceptability and safety of components are the most important criteria which should be considered in the formulation of NEs with appropriate characteristics. Appropriate mixture of oil, surfactant, and co-surfactant could result in a wide and efficient NE area (38). 

Surfactants are adsorbed at water/oil interface, resulting in a reduction in the interfacial tension to a very small value and could produce a film around the droplets with a proper curvature at the interface (41, 42). However, for the formation of a NE, a single surfactant is rarely able to provide a low interfacial tension and therefore, the addition of a co-surfactant is usually necessary. It has been reported in the literature that without a co-surfactant, an extremely inflexible surfactant film may form, leading to the production of nanoemulsions in very limited range of component concentrations (41, 42). Therefore, co-surfactant molecules (such alcohols with short and medium chain length) are frequently used to further decrease the interfacial tension, increase the fluidity of the interfacial film allowing various curvatures (43-47), reduce the bending stress of the boundary surface causing spontaneity of emulsification, lowering of size and polydispersity of droplets (44, 48).

The weight ratio of surfactant/co-surfactant has also been reported to have an important impact on size distribution, position, and the extent of NE area (42, 49, and 50). In this investigation, R_sm_ of 1:1, 1:2, and 2:1 was selected to evaluate the effect of increasing/decreasing concentrations of surfactants and co-surfactants.

**Figure 5:**
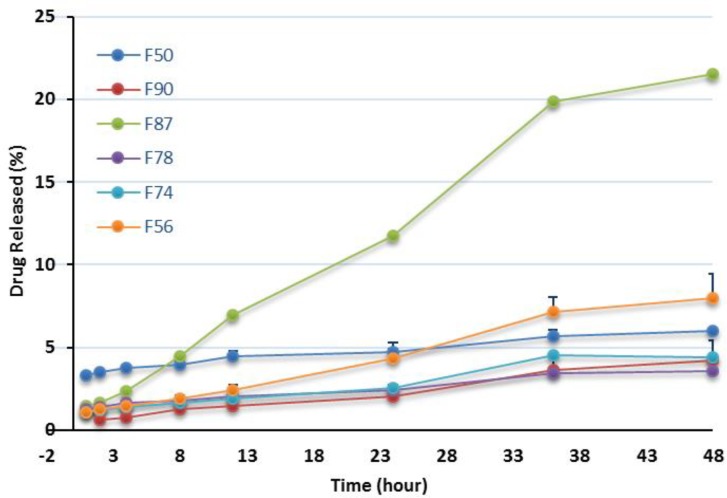
*In-vitro* release profiles of RAP-loaded nanoemulsions in the presence of oleic acid in water containing 0.05% w/v Tween 80 at 37 °C (n = 3).

**Figure 6:**
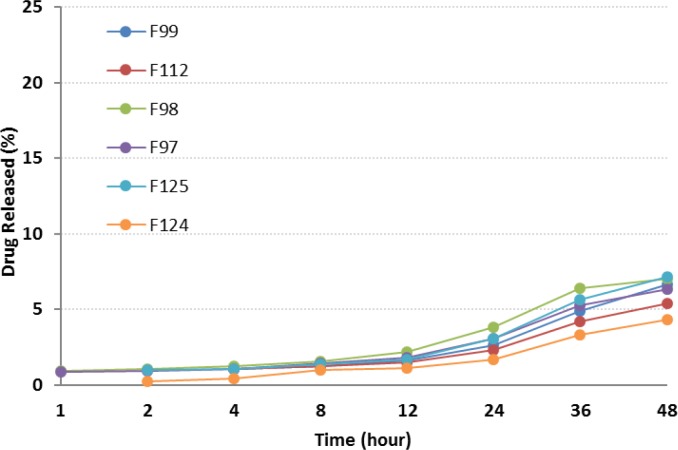
*In-vitro* release profiles of RAP-loaded nanoemulsions in the presence of Capryol^TM^ 90, in water containing 0.05% w/v Tween 80 at 37 °C (n = 3).

**Figure 7:**
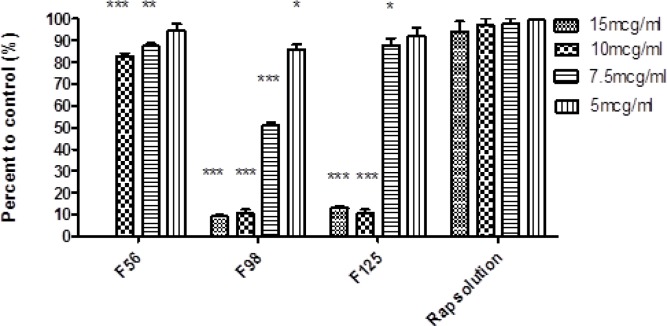
Cytotoxic effect of nanoemulsions containing rapamycin on SKBR-3 cell line measured by MTT test (48 h). 15 mcg/mL = 600 µL NE/400 µL cell culture medium; 10 mcg/mL = 400 µL NE/600 µL cell culture medium; 7.5 mcg/mL = 750 µL NE/250 µL cell culture medium, 5 mcg/mL = 500 µL NE/500 µL cell culture medium (Mean ± SD; n = 3; ^*^p < 0.05, ^**^p < 0.01, ^***^p < 0.001).

**Figure 8:**
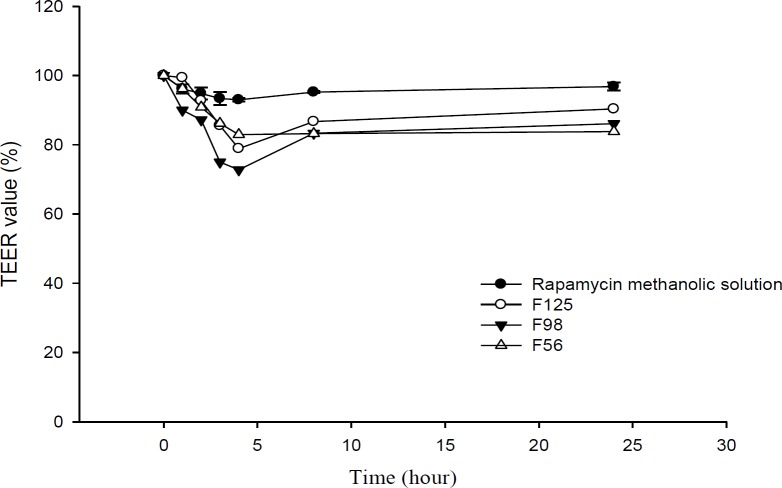
TEER values of Caco-2 monolayer, after the addition of RAP-loaded nanoemulsions and methanolic solution over a period of 24 h (n = 3).

**Figure 9:**
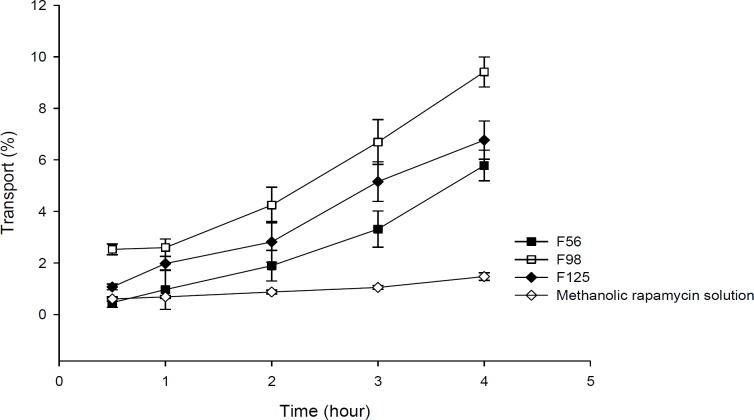
Comparison of apical to basolateral transport of RAP-loaded nanoemulsions and methanolic solution across Caco-2 cell monolayer (n = 3).

**Figure 10:**
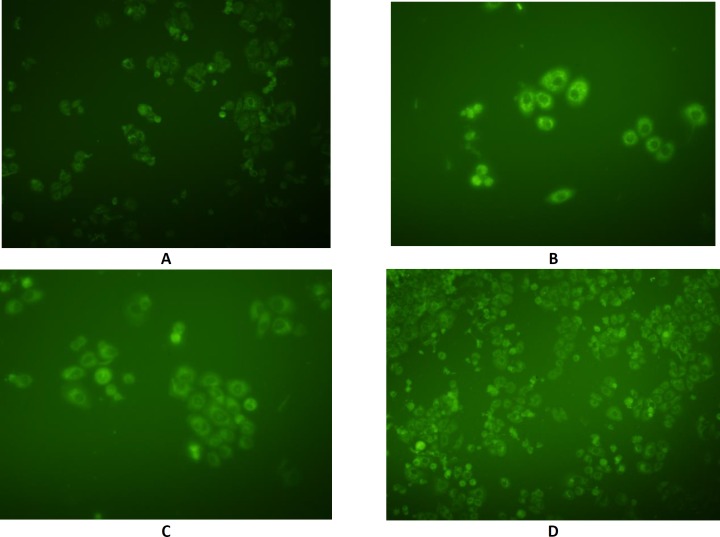
Fluorescent images of SKBR-3 cells incubated for 6 h with methanolic solution of coumarin. A) control; B) Formulation F125; C) Formulation F56; and D) Formulation F98.

**Table 1:** Composition of blank nanoemulsions, prepared at fixed total surfactant concentration of 40% w/w, selected for RAP loading.

**Formulation**	**Component**	R_sm_	**Oil ** **(wt%)**	**Water ** **(wt%)**	**Result** *
F50	OA/T20/*iso*-prop	1:2	10.07	49.93	√
F51	OA/T80/*iso*-prop	2:1	14.82	45.18	-
F56	OA/T20/PG	1:2	10.24	49.76	√
F64	OA/T80/Tr	1:1	8.98	51.02	-
F74	OA/Crem/*iso*-prop	1:2	10.00	50.00	√
F75	OA/Crem/*iso*-prop	2:1	8.16	51.80	-
F78	OA/Crem/Tr	2:1	8.58	51.42	√
F87	OA/Lab/*iso*-prop	2:1	8.58	51.42	√
F90	OA/Lab/Tr	2:1	8.58	51.42	√
F97	Cap/T20/*iso*-prop	1:1	15.24	44.76	√
F98	Cap/T20/*iso*-prop	1:2	9.40	50.60	√
F99	Cap/T20/*iso*-prop	2:1	8.98	51.02	√
F100	Cap/T20/Tr	1:1	21.90	38.10	-
F105	Cap/T80/PG	2:1	15.66	44.34	-
F111	Cap/T80/*iso*-prop	2:1	15.24	44.76	-
F112	Cap/T80/Tr	1:1	14.82	45.18	√
F121	Cap/Crem/*iso*-prop	1:1	15.66	44.34	-
F122	Cap/Crem/*iso*-prop	1:2	2.14	57.86	-
F123	Cap/Crem/*iso*-prop	2:1	2.16	57.84	-
F124	Cap/Crem/Tr	1:1	6.90	53.10	√
F125	Cap/Crem/Tr	1:2	1.90	58.10	√
F126	Cap/Crem/Tr	2:1	1.90	58.10	-

T20: Tween 20; T80: Tween 80; Lab: Labrasol ; Crem: Cremophor  RH40; *iso*-prop: *iso*-propanol; Tr: Transcutol  P; PG: propylene glycol; OA: oleic acid; Cap: Capryol^TM^ 90.

* Selected for drug loading.

**Table 2:** Droplet size and PDI values of RAP-loaded nanoemulsions (n = 3).

**Formulation**	**Z-Average (nm)**	**PDI**
F50	39.53	0.378
F56	64.36	0.449
F74	90.39	0.491
F78	26.85	0.584
F87	53.24	0.296
F90	47.49	0.349
F97	72.61	0.342
F98	74.86	0.256
F99	59.69	0.453
F112	77.25	0.483
F124	100.28	0.482
F125	100.2	0.284

**Table 3:** Mean cumulative percent of RAP released from nanoemulsions after 48 h(Mean ± SD; n = 3).

**Formulation**	**Cumulative release (%)**
F50	6.0 ± 0.22
F56	8 ± 1.47
F74	4.4 ±0.15
F78	3.6 ± 0.07
F87	21.5 ± 1.26
F90	4.3 ± 1.21
F97	6.3 ± 0.36
F98	7.0 ± 0.19
F99	6.7 ± 0.15
F112	5.4 ± 0.13
F124	4.3 ± 0.06
F125	7.1 ± 0.07

**Table 4:** Results of droplet size, PDI and drug content analysis of nanoemulsions, stored at various temperatures at the end of 9 and 12 months

**Formulation**	**4 °C**		**25 C**		**40 C**	**Drug content (%)**	
	**Size (nm)**	**PDI**	**Size (nm)**	**PDI**	**Size (nm)**	**4 C** *	**25 C** **
F56	44.4 ± 0.8	0.223	39.7 ± 0.68	0.360	phase separation	80.8 ± 0.44	4.47 ± 2.41
F87	41.9 ± 1.25	0.376	48.1 ± 1.15	0.203	phase separation	75.5 ± 2.67	6.94 ± 0.03
F98	45.3 ± 0.55	0.291	45.3 ± 0.26	0.233	79.2 ± 3.87	88.5 ± 0.24	52.94 ± 0.11
F125	101.2 ± 4.55	0.354	96.5 ± 1.36	0.269	137.8 ± 2.36	77.2 ± 0.61	57.54 ± 0.18

* 9-month storage,

** 12-month storage.

**Table 5:** Apparent permeability coefficients of rapamycin-loaded nanoemulsions and methanolic solution.

**Formulation**	**P**_app_ ** (× 10** ^-6^ ** cm/sec) ± SD**
F56	3.6 ± 0.36
F98	5.8 ± 0.35
F125	4.2 ± 0.45
RAP methanolic solution	0.9 ± 0.09

*Solubility*

Drug solubility in oil phase is a main prerequisite for the production of a stable NE formulation (26, 43). As a result of low solubility, many formulations would face with precipitation before in-situ solubilization (39). For the production of a stable NE formulation, solubility of drug in oily component is usually needed to be determined. The higher drug solubility in oil phase, the less need for incorporation of surfactants and co-surfactants (51, 52). Solubility measurement showed that RAP’s solubility in Capryol^TM^ 90 and oleic acid was 3.14 ± 0.02 mg/mL and 37.53 ± 0.23 mg/mL, respectively, which denote the potential of these oils for RAP solubility.

*Particle size and polydispersity index*

Average particle size and size distribution of RAP-loaded NEs were evaluated by dynamic light scattering technique. PDI was also determined to provide information about the deviation from the mean size. PDI is a measure of droplet size uniformity. The higher the polydispersity, the lower the uniformity of the particle size in the formulation. Table 2 represents the results of size and PDI analysis. As can be seen, nearly in all systems, the average size of the NE droplets was less than 100 nm. Formulation F78 containing oleic acid/Cremophor^®^ RH 40/Transcutol^®^ P at the R_sm_ of 2:1 yielded a nanoparticle diameter of 26.85 nm with a PDI of 0.584, whereas F124 containing Capryol^TM^ 90/Cremophor RH40/Transcutol^®^ P at the R_sm_ of 1:1 showed a nanoparticle diameter of 100.28 with a PDI of 0.482.

Although small differences were observed, however, all of the formulations investigated were composed of nano range droplets (≤100 nm). PDI is a measure of droplet size uniformity in the formulation. As can be seen in Table 2, most NEs selected for the in-vitro release study, showed PDI values less than 0.5, with the minimum value of 0.256 (in case of F98). 

*In-vitro release studies*

In this investigation, dissolution studies were carried out to compare the RAP release pattern from the NEs and confirm the release of the drug in an adequate manner. As mentioned earlier, the release behavior was studied in water having 0.05 % w/v of Tween 80 at 37 °C and the release percentage of the drug was plotted vs time up to 48 h. The release patterns of RAP from the NEs listed in Table 2 are shown in Figures 5 and 6 and the corresponding data obtained after 48 h are presented in Table 3. As illustrated from the plots for oleic acid-based NEs, except for F87, the profiles generally exhibited a relatively constant slow RAP release within 48 h, presenting a typical sustained drug release. None of the formulations exhibited complete drug release after 48 h; however, the change of release rate was found to be dependent upon the components existing in NEs. The highest and lowest releases were observed for F87 and F78 formulations, respectively. Statistical analysis revealed no significant difference in the release profile of F74, F78, and F90 (*p* > 0.05) as compared with marketed product, Rapamune^®^, with 1.2 and 2.1% drug release within 24 and 48 h, respectively. It should be noted that the release from Rapamune^®^ was observed to be incomplete which may be attributed to the gradually developing opacity of the medium, probably due to the precipitation of the drug. 

Plots in Figure 6 depict the release profiles from NEs, when oleic acid was replaced by Capryol^TM^ 90. F125 and F124 provided the highest and lowest drug release respectively. Although a slow sustained release was observed, the incorporation of Capryol^TM^ 90 did not considerably alter the release profile of the drug from NEs. After 24 h, less than 5% of the drug was released, followed by a gradual increase up to 48 h. Statistically, significant differences were observed between RAP release from all formulations and Rapamune^®^ after 24 and 48 h.

Generally, the release of RAP from NEs was found to be slightly higher when compared to Rapamune^®^. Several key factors should be considered regarding the drug delivery potential of NEs, including droplet size and polydispersity, viscosity and drug solubility in the internal oil phase. The enormous interfacial region formed by the presence of nanosized droplets could improve the solubility of a poorly soluble drug, as well as permitting faster drug release rate, and would consequently have an impact on the transport of the drug (26, 29, 39, and 53). 

In this study it was observed that RAP was generally released from NEs very slowly. No burst effect was seen and the percentage of drug release was less than 10% (except in one case). Drugs with lipophilic character, like RAP, are preferably solubilized in the oil phase of o/w NEs. The capability of maintaining these drugs in the solubilized form provides a reservoir for their sustained release. The prolonged *in-vitro* release of the drug observed could be explained by considering the partitioning of the drug towards the oil and this fact that its distribution through the oil core and interface is influenced through the aqueous media. Rouf and his co-workers developed and characterized a liposomal system for delivery of RAP and evaluated its anti-proliferative effect on MCF-7 cells as the breast cancer cell line. They observed that the percentage of drug release was very slow, with no burst effect, and in the vicinity of 10% after 24 h. They suggested that the drug molecules be located in the bilayer, not on the surface of the liposomes, as expected for a lipophilic drug (16).

*Zeta potential determination*

Zeta potential of F56, F87, F98, and F125 formulations were measured and found to be -0.03 ± 0.01, 1.28 ± 0.036, 1.01 ± 0.07, and 0.09 ± 0.007 mV respectively. Zeta potential, as a measure of charge interactions between the particles, can affect the stability of NE droplets. It has been reported that as the electrostatic repulsive forces increase, the possibility of the coalescence decreases (53, 54). The greater positive or negative zeta potential (net charge of droplets), leads to greater stability of the dispersion. 

*Stability tests*

In contrast to macroemulsions that are kinetically stable and show eventually phase separation, NEs are considered as systems with high thermodynamic stability, with no coalescence and phase separation. The selected NEs in this research were subjected to thermal stability and their drug content, size of droplet and PDI were monitored after storage at 4, 25, and 40 °C for 9 months***. ***The results are shown in Table 4. At 25 °C, a decrease in the particle size was observed which might be related to good particle stabilization. The same trend was seen for the systems stored at 4 °C. At these temperatures, no significant change of the PDI value, phase separation and turbidity was observed up to 9 months. In general, the stability results revealed that the NEs remained homogenous without any sign of instability throughout the tests. At 40 °C, only F98 and F125 systems passed the stability tests, which was also associated with an increase in the size of droplet and PDI values. In terms of the RAP assay in NEs, analysis revealed that all formulations were capable of solubilizing at least 75% of RAP at 4 °C at the end of 9 months, whereas the decline in the content of RAP after 12 months was more than 90% and 40% in oleic acid and Capryol^TM^ 90-mediated NEs, respectively.

*Cytotoxicity assay*

The cytotoxic effect of RAP-free NEs was evaluated by MTT test on SKBR-3 cell line to eliminate the possible toxic properties of the blank formulations. Cell viability in each formulation was expressed as the percentage of negative control cells. The results depicted that the blank F87 was completely toxic for the cells, even after ten times dilution and therefore, this formulation was not used for further investigation. In case of other formulations, IC_50_ was found to be more than the maximum concentration investigated. The IC_50_ value of RAP methanolic solution was also found to be 50 µg/mL and at lower concentration, more than 90% of cell viability was observed (33). Figure 7 illustrates the results of MTT test on RAP-loaded NEs. Wide range of cell death was detected, from 20% for F56 (at the concentrations of 7.5 and 10 mcg/mL) to 90 % for F98 and F125 (at the concentrations of 10 and 15 mcg/mL), which was significantly different from the results obtained for RAP solution as the control (*p *< 0.001).

In general, the obtained data confirmed that the anticancer activity of RAP would change by incorporation into NEs. Comparison of the results revealed a higher cell death when oleic acid in F56 was replaced by Capryol^TM^ 90 (in F98 and F125), probably due to enhanced penetration of RAP-loaded NE droplets to the cells and/or more RAP solubilization in Capryol^TM^ 90. Kang *et al.* have studied the solubility of celecoxib (a lipophilic drug with anticancer effect) in various oils, namely tributyrin, Labrafac^TM^ and soybean oil, and developed NE systems. Their investigation on human HCT 116 colon cancer cells have demonstrated that the cellular proliferation could be inhibited more effectively by combined treatment with free drug and tributyrin emulsions. Therefore, they suggested that the enhanced anticancer effect of celecoxib by using tributyrin emulsions was possibly due to the higher capacity of solubilization (compared to the other oils), as well as the anticancer activity of tributyrin emulsion (55). Although in our investigation, there is no significant difference between the amount of the drug released from F56, F98, and F125; however, a significant increase in the cell cytotoxicity for Capryol^TM^ 90-based NEs could be attributed to more cell penetration and drug solubilization in the internal phase. 

*Transport study*

Transport studies were performed by using the changes of TEER values as indicators for cell membrane and integrity of tight junction in monolayers of Caco-2 cells. The TEER value of Caco-2 cells cultured on filters after 21 days was calculated to be 600 Ω.cm^2^, indicating the development of tight junctions and good monolayer integrity. TEER was also measured from apical to basolateral side at specific times (1, 2, 3, 4, 8, and 24 h) in the presence of RAP-NEs and methanolic solution. After treatment of Caco-2 cell monolayers with NEs, it was seen that for all three NEs, TEER decreased to around 70-80% of the initial value. The decline in TEER value after the addition of NEs to the apical side is shown in Figure 8 and the plots of apical to basolateral transport of RAP-loaded NEs and methanolic solution across Caco-2 cell monolayer is illustrated in Figure 9. Formulations containing Capryol^TM^ 90 (F98 and F125) caused more decrease in cell integrity and drug transport, in comparison with the oleic acid containing formulation (F56).

Results obtained from transport studies suggested more RAP transport when the NEs are applied, compared with the methanolic solution and are in line with the apparent permeability calculated for each formulation. It seems that as a consequence of loading of RAP (with a high molecular weight and lipophilic character) in NEs, the cell permeability increased (Table 5).

In our previous study, we demonstrated that Tween 20, as a surfactant, can cause a significant decrease in cell integrity (33). TEER reduction represents the opening of tight junctions and reduction of cell integrity (36, 56). As reported in the literature, the components of these systems could contribute to the enhancement of drug permeation from nano- or microemulsions across monolayer of Caco-2 cells by opening the tight junctions temporarily and facilitating drug transport through paracellular pathway. Doh and co-workers developed a new lipid NE system containing granisetron and evaluated its *in-vitro* permeation-enhancing effect, using Caco-2 cell monolayers (57). Their results from permeation tests in monolayers of Caco-2 cells revealed that the NEs considerably improved the permeation of drug compared to the drug powder. They suggested that the increase in permeability was probably due to the presence of lipoid E-80 as the surfactant with a profound permeation enhancing effect (57, 58). Yin *et al.* have prepared a microemulsion system of docetaxel (a substrate of P-gp) and evaluated its oral bioavailability improvement (59). In Capryol^TM^ 90/Cremophor^®^ EL/Transcutol^® ^P system, transportation of docetaxel through the Caco-2 cell monolayer (apical to basolateral direction) and the oral bioavailability were significantly improved, compared to the commercial product. Thus, they concluded that *in-vitro* absorption of docetaxel from microemulsions could be significantly enhanced by the surfactants (*i.e.*, Cremophor^®^ EL), due to the combined effect of P-gp efflux system inhibition and increased permeability (59). Permeability enhancement effect of polysorbates on human Caco-2 cell monolayer has also been studied with Lucifer yellow assay and measurements of TEER. It has been shown that polysorbates, especially polysorbate 20 (Tween 20), could alter TEER values and increase Lucifer yellow permeability significantly (60). In addition to Cremophor^®^ EL, the enhanced permeation probably results from other compounds of microemulsions. The transportation values (P_app_) of many poorly lipophilic compounds have been reported to be greatly developed by Transcutol^®^ P and its improvement of permeation through Caco-2 cell monolayer was considerably greater than PG with the same amount (59, 61). Moreover, Cremophor^®^ RH 40 could similarly inhibit P-gp efflux pumps (62-64), although it was reported that Cremophor^®^ EL inhibits P-gp efflux pumps stronger than Cremophor^®^ RH 40 (65). In addition to the above mentioned mechanism, the properties of NEs, such as the presence of nanosized droplets and the interaction between the cellular membrane and surfactants, could also enhance the drug permeation (57).

*Cellular uptake of nanoemulsions*

In order to detect the intracellular position of coumarin-6, as a hydrophobic cellular uptake indicator which could emit green fluorescence in SKBR-3 cells, fluorescence microscopy was performed using coumarin 6-loaded NEs (Figure 10). Fluorescence intensity in different cytoplasmic regions with F56, F98, F125 formulations, and coumarin methanolic solution was 57.18, 59.02, 48.1, and 29.87, respectively. The dye was associated with the oily part of nanoemulsions and its presence inside the cells could be expected to be due to the internalization of the nanoemulsion droplets inside the cells which in turn rendered the cells fluorescent. These results are in agreement with those obtained from TEER and cytotoxicity experiments.

## Conclusion

In summary, the results of the present investigation demonstrated that the developed oil-in-water NEs, composed of oleic acid and Capryol^TM^ 90 as inner oil phases, Tween 20 and Cremophor^®^ RH 40 as surfactants, were thermodynamically stable and possessed efficient solubilizing capacity for RAP. The optimized formulations showed considerable toxic activity against breast cancer cell line SKBR-3. *In-vitro* transport studies through monolayer of Caco-2 cell also revealed considerable enhancement of the drug transport. This and the previous investigation showed that the use of a nanoemulsion-based vehicle could be considered as an effective and promising strategy for the delivery of RAP from pharmaceutical and toxicological points of view, although further studies are required.
